# Sustainable development of bioepoxy composites reinforced with recycled rigid polyurethane foam for mechanical, thermal, acoustic, and electromagnetic applications in a circular economy approach

**DOI:** 10.1038/s41598-025-91273-9

**Published:** 2025-03-10

**Authors:** Vinoth Kumar Selvaraj, Jeyanthi Subramanian, S. Mouleswaran, T. R. Keshav Keerthan, Thangapandi Muneeswaran, A. Kishore Nath, M. Padmanabha Raju

**Affiliations:** 1 School of Mechanical Engineering, Vellore Institute of Technology, Chennai, Tamil Nadu 600127 India; 2 Sedaxis Advanced Materials Pvt Ltd., Chennai, Tamil Nadu 600066 India; 3 Advanced System Laboratory, Defence Research & Development Organisation, Hyderabad, India

**Keywords:** Recycling, Sustainable composites, Bio-epoxy, Net-zero carbon emission, HR-SEM, FTIR, TGA, Engineering, Materials science

## Abstract

The accumulation of polyurethane (PU) waste presents a critical environmental challenge due to the inefficiencies of traditional disposal methods like landfilling and incineration. This study introduces a sustainable approach by repurposing 99.89% pure rigid polyurethane foam granules (~ 150 µm) as fillers (5 wt.%) in bio-epoxy composites, complemented with 99.89% pure vermiculite particles (~ 10 µm) at varying concentrations (2–10 wt.%). Comprehensive characterization techniques, including high-resolution scanning electron microscopy (HR-SEM) and Fourier transform infrared spectroscopy (FTIR), were employed to evaluate the composites’ mechanical, thermal, electrical, acoustic, and electromagnetic interference (EMI) shielding properties. The study specifically measured EMI shielding effectiveness in the frequency range of 8–12 GHz. Among the formulations, sample S5 exhibited superior mechanical performance, with tensile strength (10.47 N/mm2), impact strength (0.006 kJ/cm2), and flexural strength (46.80 N/mm2). EMI analysis revealed a dielectric constant of 1.111 and shielding effectiveness of -35.24 dB, while sample S3 achieved optimal acoustic absorption (NRC 0.295). Thermal assessments showed the lowest thermal conductivity (0.141 W/mK) and a reduced burning rate (6.8 mm/min) for S5. These results highlight the viability of recycled PU foam-based composites in minimizing plastic waste and advancing net-zero carbon emission goals. Potential applications include battery enclosures, engine bay insulation, and cabin soundproofing in electric vehicles. This work establishes the novelty of integrating recycled materials into bio-epoxy matrices to address environmental challenges and create high-performance composites.

## Introduction

The pursuit of net-zero carbon emissions has garnered significant attention in recent years, emphasizing the need to minimize the carbon footprint associated with products and consumption patterns to achieve carbon neutrality. Carbon neutrality is defined as a balance between the greenhouse gases emitted into the atmosphere and those removed or offset through sustainable practices. A central focus of recent research has been on the reuse and repurposing of non-biodegradable materials, aligning with circular economy principles to advance the goal of carbon neutrality and mitigate environmental impacts.

PU is one of the most common polymers for various purposes around the globe, standing as the 6th most used polymer. Simultaneously it is concerning that 50% of the WRPU waste reaches landfills without ever going through the recycling or reuse processes. Hence it is crucial to recycle and reuse the waste produced and avoid getting it in landfills and incinerated. There are many methods of recycling PUs such as Chemical recycling, Thermochemical recycling, and Mechanical recycling^1,2^. Chemical recycling is effective in recycling all types of polymers^3^. The most used method for WRPU is glycolysis. In this process, the rigid PUs is mixed with surfactant, catalyst, phosphate, and blowing agents to form a homogeneous polyol. This mixture is then stirred at 6000 rpm for 7 s, poured into a mould, cured at 60 °C for 20 min, removed from the mould, and cured for 24 h^4^. Thermo-chemical recycling is another process for recycling WRPU foams in which there are many methods such as pyrolysis, gasification, hydrogenation, and blast furnace. Pyrolysis is a method in which WRPU is decomposed thermally to break down the chemical structure to produce hydrocarbons with a minimal waste product called char^5^. In an experimental study by N. Gama, et al. WRPU scraps were recycled using acidolysis. In this process WRPU is broken down into polyol using acids in this experiment succinic acid was used, and thus the reactive raw material can be used to produce fresh WRPU or other materials. Moreover, this process does not produce carcinogenic aromatic amines^6^. Biodegradation of PUs using microbial farms might pose a permanent solution to the environmental issues. There exist many bacterial and fungal organisms that produce chemicals that can degrade the PUs. Still, there’s no single organism that could be mass cultured to biodegrade the PUs and usually, the organisms only break down some parts of the compound^7^. The most basic method used to recycle WRPU is mechanical recycling, and it can be manufactured at high output^8^. In this process, the used WRPU in solid form is ground into powder and can directly be used for applications such as pillows and toys. It can also be further refined by secondary recycling which is grinding them into finer granules that are less than 100–125 microns in size using the two-roll mills processing. Then the WRPU granules can be used as fillers in newly manufactured PUs^9^.

At present, it is more reliable and more convenient to incorporate mechanically ground WRPU powder into epoxy resin and produce new materials. It is also more efficient since mechanically grinding the WRPU wastes in pellet form takes less time compared to any other forms^10^. Epoxy resins have high thermal and chemical resistance, good mechanical properties, and are easy to process. Some types of epoxy resins even have good electrical conductivity, self-healing properties, rapid curing time, and even recyclable^11^. Di glycidyl ether of bisphenol A is one of the most used types of epoxy resin but it depends upon fossil fuels^12,13^. As a replacement, bio-based epoxy has been widely studied in the past years^14^. The bio-resins that are widely studied are epoxy resin made with Itaconic acid, Aliphatic-based bio-resins, and bio-resin made with glycerol and sorbitol^15,16^. Aliphatic-based bio-resins have poor mechanical and thermal properties but can still be used as coatings and active diluents. One of the promising replacements is bio-based epoxy resin made with Itaconic acid. Comparing it with DI glycidyl ether of bisphenol A, itaconic acid bio epoxy has a higher epoxy value, lower viscosity, and better curing reactivity^17,18^. 2,5-furan-dicarboxylic acid also known as FDCA is a compound used to make thermoset materials and has shown to have high thermal, mechanical, and recycling properties^19^. Another type of bio-resin made with glycerol and sorbitol as cores can produce hyperbranched epoxy resin that has better viscosity, and functionality and has similar tensile and thermomechanical properties to petroleum-based epoxies^20^. Although epoxy resins have a wide range of applications, they cannot withstand compressive strength. To compensate for this disadvantage WRPU powder is incorporated into epoxy resin. This was carried out and the results showed improvement in thermal and mechanical properties of the overall compound^21^. Further, WRPU epoxy composites can be used for anti-erosion applications because of their suitable mechanical and water-resistant properties. These composites can further be strengthened using other nanomaterials such as Al_2_O_3_, nano-silica, TiO_2_, etc. for improved wear resistance and better mechanical properties^22^. WRPU and 8.75% Al powder mixed with epoxy show improved mechanical properties and low IR emissivity^23^. However, the flammability of WRPU is a concerning issue with the material’s applications.

The addition of VMT, a phyllosilicate material when mixed as a binder improves the thermal, mechanical, and acoustic properties^24^. When mixed with rigid WRPU and epoxy it shows better thermal and mechanical properties compared to pure rigid WRPU compound^25^. They could be used in brake pad linings due to their mechanical, thermal, and friction properties^26^. The total heat release and smoke decreased compared to the rigid WRPU compound^27^. In an experiment done by mixing rigid WRPU and VMT, it was observed that the samples with a high composition of VMT showed better results in the vertical flammability tests and also retained the structural integrity of the sample better than the lower compositions^28^. When Expanded VMT treated with hydrogen peroxide, is added to WRPU it decreases the overall thermal conductivity and flammability compared to a compound with the same amount of VMT that is not expanded and treated with H_2_O_2_^29^. When VMT is added to an Al metal matrix, the mechanical properties such as tensile strength and impact strength increase when 5% of wt. composition is added, and when 2% wt. composition is added, the composite has increased hardness^30^. VMT when added with other organic substances such as flax fiber and sphagnum moss shows better sound absorption properties. But flax fiber and sphagnum moss when tested for biodegradation possess the risk of being decomposed easily and losing their properties^24^. VMT when mixed with other organic compounds such as eggshell, PVC, and sawdust can even be used for radiation absorption applications^31^. When VMT is added to foam concrete, the material’s thermal conductivity decreases. But when the VMT composition is increased, and the concrete composition is decreased, there’s a sharp decrease in the compressive strength of the material^32^. VMT is also used as an acoustic material as it is porous and can also be added with other substances to improve its mechanical strength and thermal properties^33,34^.

To test a bio-based composite’s reliability, we must test various properties such as tensile test, impact testing, acoustic, dielectric, EMI shielding, thermal conductivity, and flammability. In research done with bio polyethylene and VMT, the material’s tensile property was tested and found to be increasing when the wt.% of VMT increases, reaching up to 21.93 ± 0.08 MPa when VMT is at 10% wt. composition^35^. Another research done with biobased polyethylene composites filled with expanded VMT using a rotational moulding process shows us that with the increase in the wt.% of expanded VMT, there was a decrease in the impact strength of the materials^36^. Another research was done to improve the dielectric constant of shellac using natural clays for organic field effect transistors done with VMT and it was found that VMT and halloysite when mixed with shellac at a 1:1 ratio the dielectric constant of the composites is found to be 5 and 5.3 respectively^37^. Another research where the electrical properties of epoxy-based bio-composites of sample 1 made of cotton fiber fabric and sample 2 made of cotton fiber fabric with addition to flax fiber fabric. Each sample had a reference specimen of circular shape of diameter 33 mm and a load sample with an external diameter of 133 mm. It was found that the cotton fiber in addition to flax fiber fabric had better electrical conductivity, because of the metallization quality of the flax fiber surface and the variation of the fiber volume fraction added to the specimen. Both specimens showed higher Shielding effectiveness at higher frequency electromagnetic waves ranging from 60db up to 90db^38^.

Another research where the flexible WRPU foams are coated with layers of bio-based resin alginate, chitosan, and hydroxyapatite to reduce the flammability shows that the samples are subjected to 20 mm long butane flame for 10 s shows a reduction in the flammability of the material and a decrease in self-extinguishing time for the samples when the layers of bio-based coating are increased^39^. Research done with VMT and WRPU to test the material’s flame retardancy shows an increase in the degradation temperature with an increasing amount of VMT but the amount of residue left behind also increases substantially^40^. These composites can be used in the aerospace industry as there is a requirement for EMI shielding, and noise dampening, handling high-temperature fluctuations and having less weight. They could also be used for MRI machines where the high temperature produced by the strong magnets must be handled and reduce the noise produced by the machine to provide a calm environment for the patients.

This study introduces a novel approach to waste management by repurposing recycled rigid polyurethane foam granules and vermiculite into bio-epoxy composites. The resulting materials exhibit enhanced mechanical, dielectric, EMI shielding, acoustic, and thermal properties. This sustainable solution for reducing plastic waste and promoting environmental sustainability offers a promising avenue for the development of new materials.

## Materials and methods

### Materials

The type of epoxy resin chosen is a bio epoxy manufactured by Ecopoxy, Canada. The bio epoxy is chosen for its fast curing and ability to fabricate samples of a wide range of sizes. The hardener was purchased from the same company. The waste rigid polyurethane was acquired from ASL DRDO, Hyderabad. The vermiculate is purchased from DK Enterprise, Chennai, India. The details of fillers are shown in Table 1.

**Table 1:** Details of fillers reinforced in bio-based epoxy.

Filler	Size (µm)	Density	Specific heat	Melting point
PU	100–150	50–170 kg/m^3^	1,200–1,800 J/kgK	65–120 °C
VMT	10–50	64–160 kg/m^3^	%1.%2-%3.%4 J/kgK	1240 – 1430 °C

### Fabrication

**Fig. 1:**
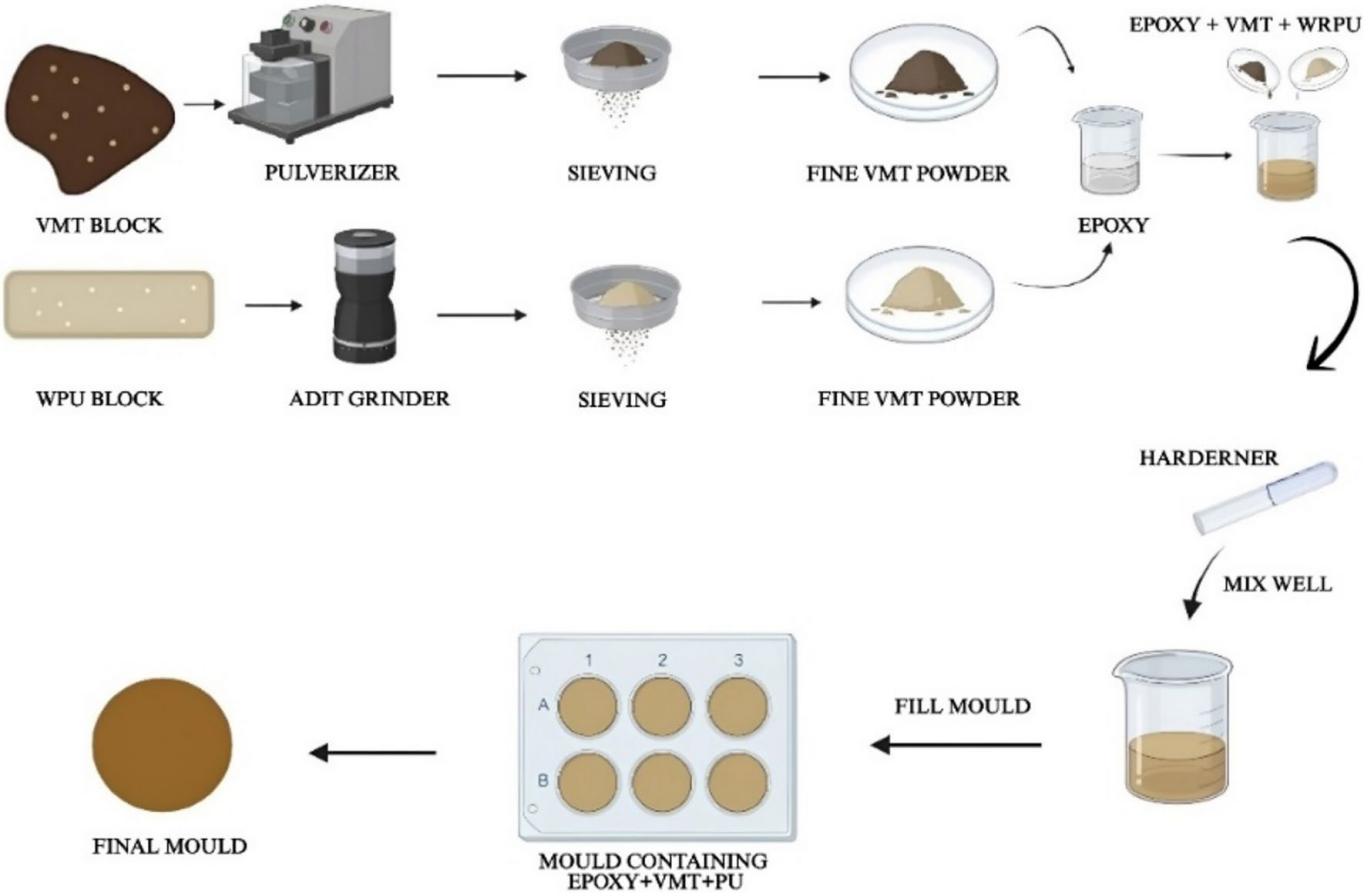
Manufacturing process of fillers-instigated bio-based epoxy.

The waste rigid polyurethane foams are grinded using an ADIT grinder and the vermiculate particles are grinded using a pulverizer and they are turned into smaller particles. The WRPU and vermiculate particles are then sieved using a sieving machine for 20–30 min to obtain particles of the same size. The WRPU powder procured is around 150–200 µm and the vermiculate obtained is around 10 µm. The moulds made for the samples are made with silicone mould manufactured by Chemzest Techno Products PVT LTD, Chennai, India. The silicone is poured into testing models that are 3d printed with 10 mm precision and left to cure until hardened. The silicone mould is then removed and used for the fabrication of the samples. The S0 sample (0 wt.% filler) is made of pure epoxy to analyze the effects of the fillers in the epoxy matrix. The WRPU powder is then measured for 5 wt.% for every sample and the vermiculate is measured for 2,4,6,8 and 10 wt.% for S1, S2, S3, S4, and S5 samples respectively. The remaining wt.% is then filled with epoxy. The epoxy and the WRPU powder are mixed for 10 min at 50 rpm by mechanical stirring then the vermiculite is added based on the sample wt.%. The epoxy mixture is then again mixed for 20 min at 50 rpm by mechanical stirring. The hardener is then added as per a 2.5:1 ratio for each sample and mixed at 50 rpm for 5 min. The composite is then poured into the silicone moulds and cured. The samples are then retrieved from the moulds and then tested to check the various properties and the effect of the filler composition on those properties. The manufacturing process of fillers-instigated bio-based epoxy is shown in Fig. 1.

### Mechanical testing

#### Tensile test

The tensile test is carried out in Shimadzu Universal testing machine at 24 $\pm$ 1 $^\circ{\rm C}$ temperature and 50 $\pm$ 2% relative humidity. The samples were prepared as per ASTM 3039 (250 × 25 × 2.5 mm^3^) standards and analyzed at 2 mm/min.

#### Flexural test

The flexural test is carried out in Shimadzu Universal testing machine at 24 $\pm$ 1 $^\circ{\rm C}$ temperature and 50 $\pm$ 2% relative humidity. The samples were fabricated as per ASTM D7264 (155 × 13 × 4 mm^3^) standards.

#### Impact test

The impact test is carried out in J Tech Instruments impact testing apparatus. The samples are prepared as per ASTM D256 (75 × 10 × 10 mm^3^) standards. The strength testing is followed as per Izod testing standards.

### Electrical testing

#### Dielectric constant

The dielectric test is carried out in Asico instruments. The samples are prepared as per ASTM D150 standards. The test uses a dielectric constant (DC) apparatus, a test capacitor (TC), and a variable gang capacitor (VGC). First, the VGC’s initial reading (C1) is recorded. The TC is then connected after placing the sample between its movable plates. The resonance point (C2) is found by adjusting the variable resistance. After removing the sample, the resonance point (C3) is determined.

1 $$K=\frac{C1- C2}{C1 - C3}$$

The DC of the material is determined using Equation $1$.

2 $$\delta =\frac{c}{\omega \surd {\varepsilon }_{r}}$$

The Skin depth of the material is determined using Equation $2$.

3 $$\sigma =\frac{1}{{\mu }_{0}{\mu }_{r}{\delta }^{2}\omega }$$

The Conductivity of the material is calculated using Eq. 3.

### Electromagnetic testing

#### EMI shielding

The EMI shielding test is carried out by an EMI test setup with model number N5230A PNA-L. The samples were fabricated as per ASTM D4935 specifications (50 × 50 × 7 mm^3^).

### Acoustic testing

The acoustic property is tested for varying filler composition. The samples are made with the standards of ASTM E1050-19 (100 mm diameter and 20 mm thickness). The samples are tested using impedance tubes.

### Thermal testing

#### Thermal conductivity

The thermal conductivity of the samples is tested as per the ASTM D5470 (25 × 25 × 5 mm^3^) standard. Thermal conductivity is measured using a C-Therm Trident from MTPS guard ring technology in IITM.

#### Flammability test

The flammability tests are carried out by the standard UL 94 vertical burning test ASTM D3801. The sample dimensions were 125 × 13 × 3.2 mm^3^. The presto flammability tester is used to test a five-sample set with variable VMT (2–10 wt.%) and 5 wt.% WRPU.

## Results and discussion

### HR-SEM characterization

**Fig. 2:**
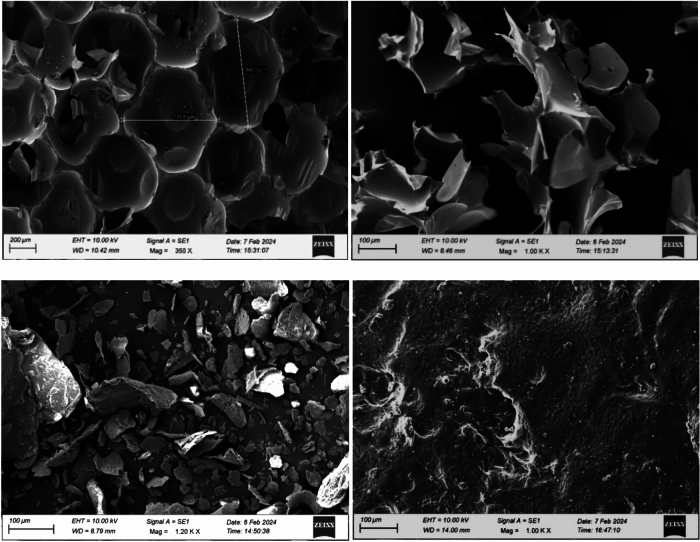
HR-SEM of (**a**) RPU, (**b**) WRPU, (**c**) VMT, (**d**) Bio-based epoxy with fillers.

The micro surface morphology and structure of the VMT, WRPU, and epoxy with fillers were observed using HR-SEM (High-Resolution Scanning Electron Microscopy), As shown in Fig. 2. Before scanning, the samples are gold-coated. In Fig. 2(a), RPU’s closed pores are uniformly packed and size range from 700 to 800 µm and Fig. 2(b) shows the structure morphology of WRPU from 1.00KX magnification. The sharp, brittle pieces of polyurethane structure are found after grinding. The average particle size is from 80–140 µm. Figure 2(c), shows how VMT can form with a flake-like structure with particles that range in size from 20–100 µm. The separation between each particle shows a uniform distribution of VMT. Finally, in Fig. 2(d), it is observed there is a homogeneous distribution of RPU and VMT in the layer of bio-epoxy which forms an undulation.

### FTIR characterization

**Fig. 3:**
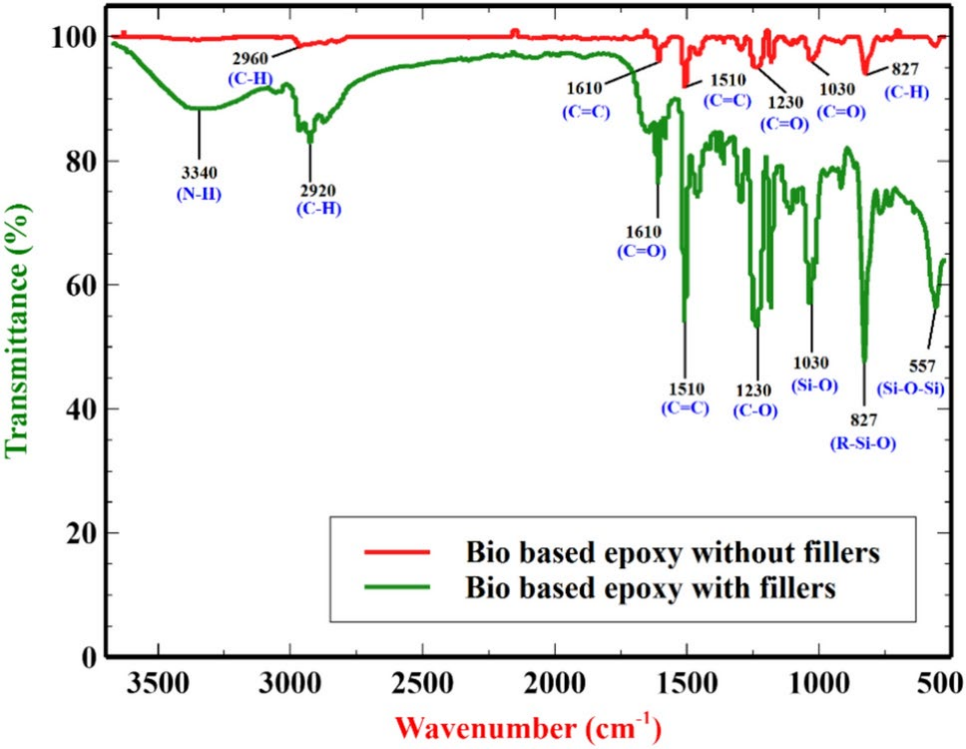
FTIR graph for Bio-based epoxy without and with fillers.

The chemical structure of neat epoxy and epoxy with fillers (PU, VMT) are characterized by FTIR equipped with single-bound ATR attachment. The samples were examined between 500 to 4000 cm^−1^ wavelengths. The FTIR spectra of each bio-based epoxy sample, both with and without fillers, were obtained Fig. 3. The bio-based epoxy sample without fillers has a high wavenumber at 2960 cm^−1^, which corresponds to the stretching of the C-H group, and at 1610 and 1510 cm^−1^, which exhibits C = C stretching vibration in aromatic. The peak at 1230 cm^−1^ represents asymmetrical aromatic C = O stretching. The symmetrical aromatic C = O stretch has a peak wavenumber of 1030 cm^−1^ and C-H out-of-plane deformation in aromatic stretching is represented by 827 cm^−1^^41^. The bio-based epoxy samples with fillers show the highest wavenumber at 3340 cm^−1^ was corresponding to N–H stretching and the wavenumber 2920 cm^−1^ is ascribed to the C-H stretching. The absorption crest at wavenumber 1610 cm^−1^ is correlated with the stretching vibration of the urethane C = O bond. The stretching band of C-N wavenumber from 1690 cm^−1^ to 1620 cm^−1^ is observed. The stretching band at 1140 is corresponding to the C-O group of ether group in WRPU foam. The stretching at 3660 to 3080 cm^−1^ is related to the O–H group in the interlayer of water molecules located at 3340 cm^−1^. The peaks detected at 1030, 827, and 557 cm^−1^ were characterized by the stretching vibration of Si–O, R–Si-O (R = Mg, Fe, Al, etc.) and the bending vibration of Si–O-Si^42^.

### TGA (Thermal gravimetric analysis)

**Fig. 4:**
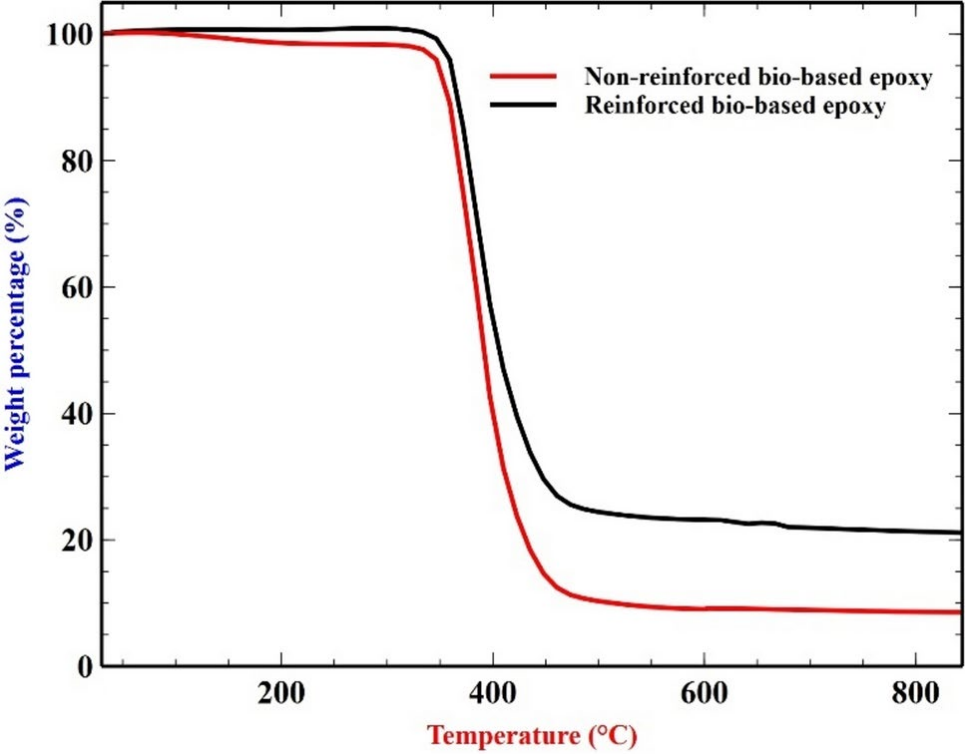
Thermal gravimetric analysis.

The thermal gravimetric analysis (TGA) is performed using SDT Q600, TA Instruments, USA at a heating rate of 10** °C**/min. A sample of about 10 mg is placed in an Al crucible and N_2_ gas is supplied at 50 mL/min to increase the temperature from 25 °C to about 850 °C. From Fig. 4 it is evident that the reinforced sample performs better than the non-reinforced sample. The non-reinforced sample decays around 340 °C while the reinforced sample shows slight improvement of around 370 °C. The non-reinforced sample continued to decay to 90% weight loss in the range of 340℃−440 °C while the reinforced sample decays only to 75% weight loss before it stabilizes and shows no significant weight loss over a large temperature. A 15% difference of weight reduction is achieved from the addition of filler materials.

### XRD analysis

**Fig. 5:**
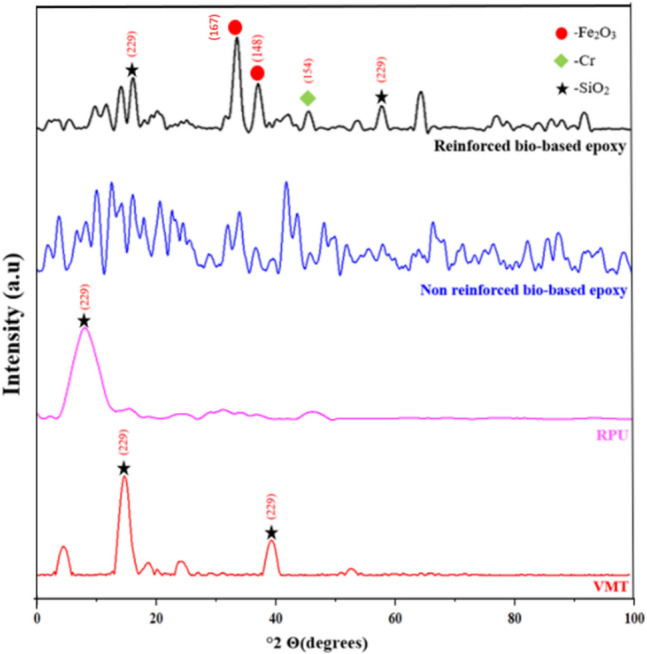
XRD of Bio-based epoxy with & without fillers.

The chemical composition of the samples and the fillers were observed using powder XRD analysis. X-ray is generated by the transition of electrons when bombarded with high-speed electrons and by analyzing the pattern generated by the diffraction, we can find the phase and microstructure of the material^43^. Figure 5 represents the XRD analysis of samples and the fillers respectively. Components such as iron oxide and silicon oxide were identified in both the samples and the fillers^44^ .This indicated that the vermiculate originated from micaceous minerals that were weathered^45^.The peak at around 20 degrees for RPU is due to its powdered structure which indicated that the structure is destroyed, and the structure becomes amorphous^46^. The crystallographic structures were also found to be 167 for Fe_2_O_3_, 229 for Cr and 154 for SiO_2_.

### Mechanical testing

#### Tensile test

**Fig. 6:**
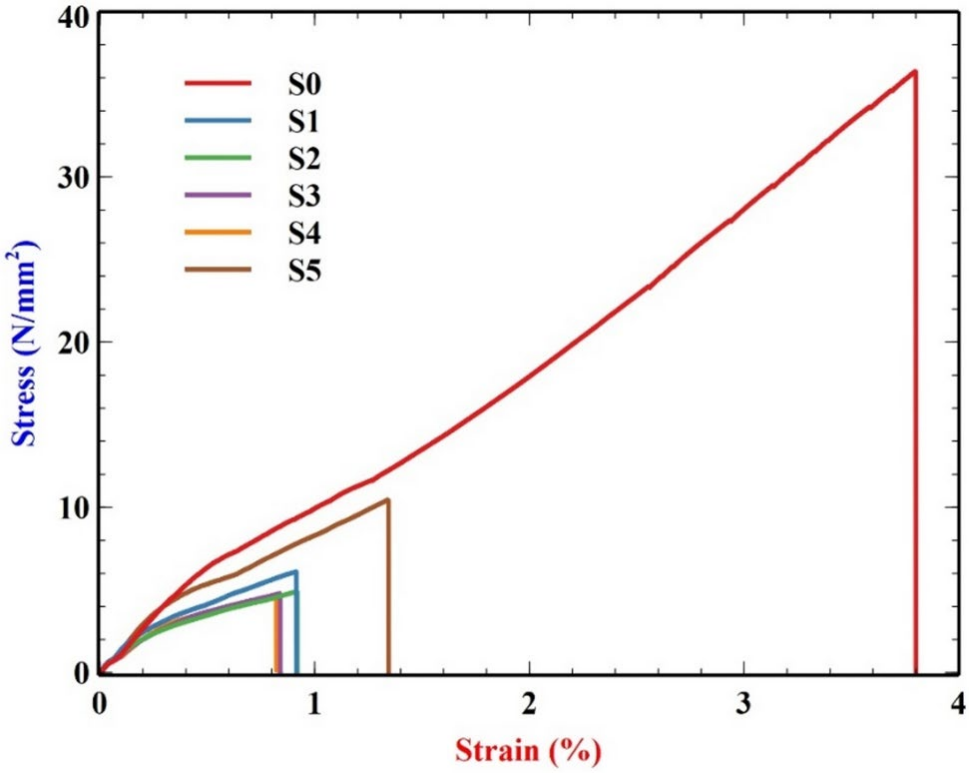
Stress–Strain graph for tensile test.

Table 2 and Fig. 6 represent the maximum stress and max strain percent from the tensile test of several specimens with varying mass percentages of VMT, ranging from 2 to 10%. The specimen’s maximum load-bearing capacity and maximum deformation under tensile loading were determined, and the trend was shown on a graph to compare it to findings from other studies. The specimen with 10% VMT fiber loading was found to withstand a maximum load of 1171.13 N and a maximum deformation of 1.34354%. In contrast, the specimen with 8% VMT fiber loading could withstand the least load of 527.708 N and deformation of 0.82106%. Initially, there is a decrease in stress from S0 and S1 this is due to the elasticity of pure epoxy without any fillers which can be seen as the maximum strain percent in the S0 component is higher than in other samples, in addition, the 10% VMT has more load bearing capacity and withstands maximum loading without deformation^47^.

**Table 2:** Experimental values of tensile stress and strain.

Samples	Max. force (N)	Max. stress (N/mm^2^)	Max. strain (%)
S0	4072.13	36.4078	3.79918
S1	683.641	6.11224	0.91440
S2	549.531	4.91320	0.91906
S3	535.925	4.79155	0.83904
S4	527.708	4.71808	0.82106
S5	1171.13	10.4707	1.34354

##### Impact test

**Fig. 7:**
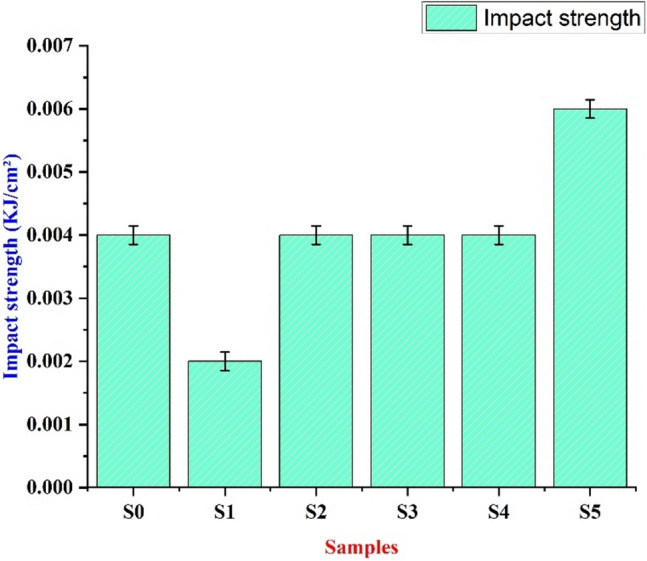
Impact strength for different bio-epoxy composites.

An impact test is conducted on several specimens with varying VMT mass percentages from 2 to 10%. From Table 3 and Fig. 7, we can infer that in sample S1 there is a decrease in the impact strength of the specimen when compared to the S0 epoxy sample. This is due to the elasticity of the pure epoxy sample. However, as the VMT amount increases, the impact strength of the specimen is gradually increased. At the end, the impact strength of the S5 sample of 10% VMT and 5% recycled WRPU has higher impact strength compared to the S0 sample. The reason for the drop in impact strength can be correlated to the elasticity of pure epoxy samples^48^.

**Table 3:** Impact Test.

Test No	Material Name	Length (cm)	Breadth (cm)	Area (cm)	Falling Angle (°)	Rising Angle (°)	Energy Loss (kJ)	Energy (kJ)	Impact strength (kJ/cm^2^)
1	**S0**	10.000	10.000	100.000	104.0	102.0	0.000	0.400	0.004
2	**S1**	10.000	10.000	100.000	104.0	103.0	0.000	0.200	0.002
3	**S2**	10.000	10.000	100.000	104.0	102.0	0.000	0.400	0.004
4	**S3**	10.000	10.000	100.000	104.0	102.0	0.000	0.400	0.004
5	**S4**	10.000	10.000	100.000	104.0	102.0	0.000	0.400	0.004
6	**S5**	10.000	10.000	100.000	104.0	101.0	0.000	0.601	0.006

##### Flexural test

**Fig. 8:**
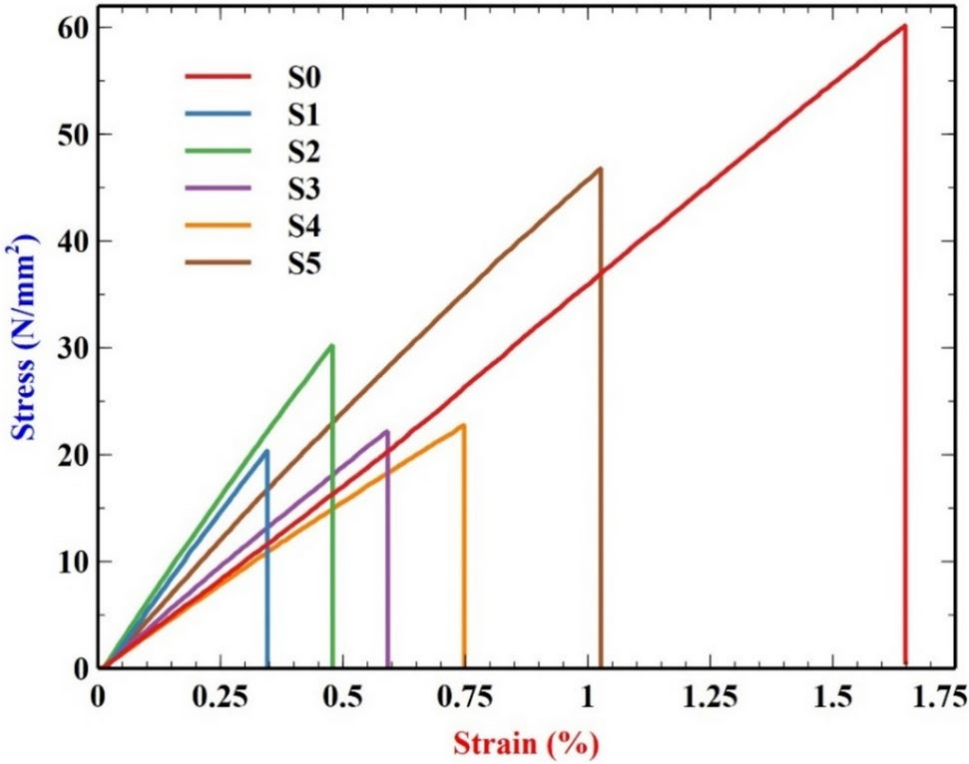
Stress–Strain graph for flexural test.

The samples from 0% filler material to 10% filler material are tested for flexural stress and strain. From Table 4 and Fig. 8, we can see that the S0 performed better than all other samples and this is due to the nature of epoxy. Pure epoxy deforms more than that of the other samples, so it has a higher strain percentage compared to other samples. As seen in other mechanical tests, sample 0 performed better than other samples, but since it deforms more than other samples, it cannot perform better than the samples with fillers. Even though the addition of the filler material decreases the flexural strength, it is to be noted that the increase in the filler material from 2 to 10% gradually increases the flexural strength^49^.

**Table 4:** Stress and Strain table.

Samples	Max. force (N)	Max. stress (N/mm^2^)	Max. strain (%)
S0	77.0330	60.1821	1.64798
S1	26.0830	20.3773	0.34581
S2	38.7510	30.2742	0.47894
S3	28.4195	22.2027	0.59150
S4	29.1268	22.7553	0.74766
S5	59.8987	46.7959	1.02654

### Electrical testing

#### Dielectric test

**Fig. 9:**
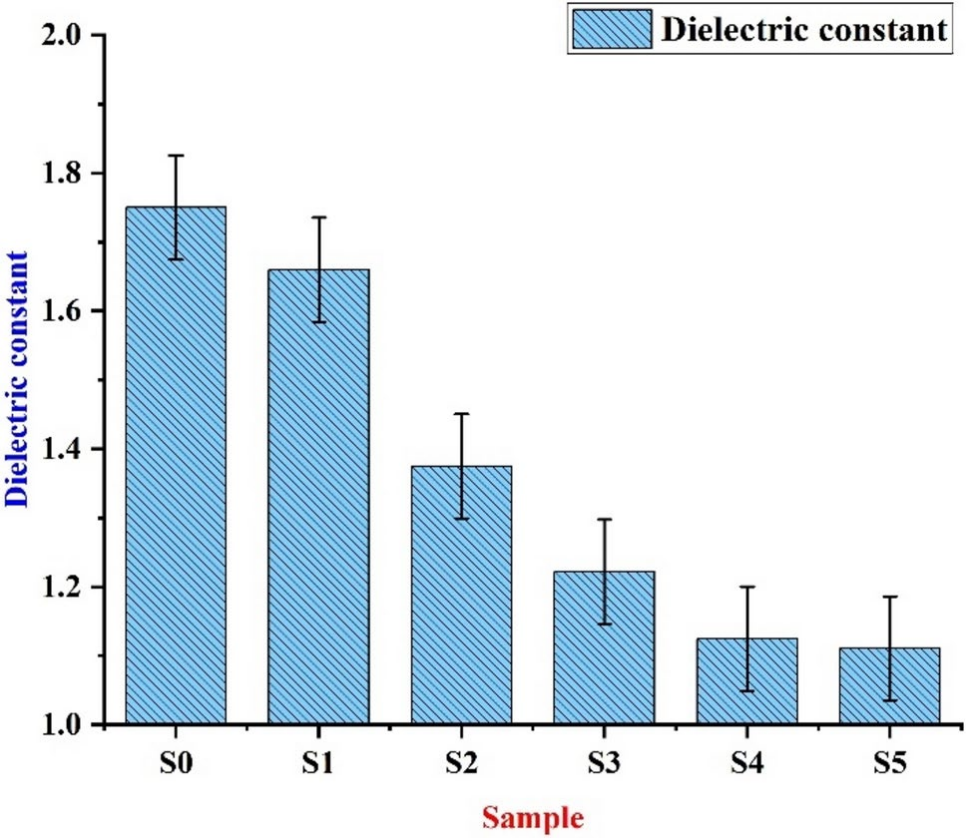
Dielectric constant for different bio-epoxy composites.

By employing a dielectric constant device, we calculate the dielectric constant (DC) by detecting the fluctuating capacitance of the test samples and standard specimens under resonance with 10 MHz . First, the VC of the test sample is determined. Subsequently, the conventional capacitor values with and without dielectric material are noted. Likewise, the same procedure is used for the same samples once more. The dielectric constant of the samples is calculated by Equation $1$ and is shown in Table 5. From Table 5, and Fig. 9, it can be inferred that the specimen without added fillers (S0) has the highest dielectric constant of 1.75. And the specimen with 10% VMT and 5% WRPU has the least dielectric constant of 1.11. As we know it, materials with less dielectric constant values have higher resistance and function well as insulators. Consequently, the test sample (S5) is 45% more insulating than the sample (S0). The skin depth of the specimen is calculated by Equation 2 and it varies from 3.61 m (S0) to 4.53 m (S5), indicating how deeply electromagnetic waves penetrate before significant attenuation. S0 sample has thinner skip but a clear trend emerges where higher skin depth corresponds to better EMI shielding. This behaviour is likely influenced by VMT moisture retention and dielectric loss, which enhance attenuation and WRPU role on its structure; open-cell variants of porosity and moisture absorption, further affecting dielectric properties. The epoxy matrix provides structural stability and has a higher dielectric constant but specimen’s overall shielding performance is largely driven by the VMT and WRPU ratio and their interaction with electromagnetic waves. The conductivity is calculated by Equation 3 and it decreases from S0 to S5 primarily due to the reduction in the dielectric constant and the increasing presence of insulating materials like VMT and WRPU.

**Table 5:** Dielectric constant.

Samples	C1	C2	C3	Dielectric constant	Skin depth(m)	Conductivity(S/m)
S0	100	86	92	1.75	3.6	0.000972
S1	100	90	94	1.66	3.71	0.000922
S2	100	89	92	1.375	4.07	0.000764
S3	100	89	91	1.2222	4.32	0.000679
S4	100	91	92	1.125	4.50	0.000625
S5	100	90	91	1.111	4.53	0.000617

**Table 6:** Experimental values of EMI shielding.

Samples	Transmission coefficient	Absorption coefficient	Shielding effectiveness absorption (SE_abs_) (dB)	Total electromagnetic interference shielding (SE_Total_) (dB)
S0	18.576 ~ 175.032	−20.96 ~ −192.70	−8.91 ~ −9.960	−12.69 ~ −22.431
S1	129.276 ~ 1752.259	−131.66 ~ −1769.92	−17.34 ~ −19.965	−21.12 ~ −32.435
S2	148.596 ~ 1672.81	−150.98 ~ −1690.47	−17.94 ~ −19.763	−21.72 ~ −32.234
S3	148.108 ~ 3180.96	−150.49 ~ −3198.62	−17.93 ~ −22.555	−21.71 ~ −35.025
S4	146.168 ~ 2846.222	−148.55 ~ −2863.88	−17.87 ~ −22.072	−21.65 ~ −34.542
S5	140.659 ~ 3346.622	−143.05 ~ −3364.28	−17.71 ~ −22.775	−21.48 ~ −35.246

### Electromagnetic testing

#### EMI shielding

**Fig. 10:**
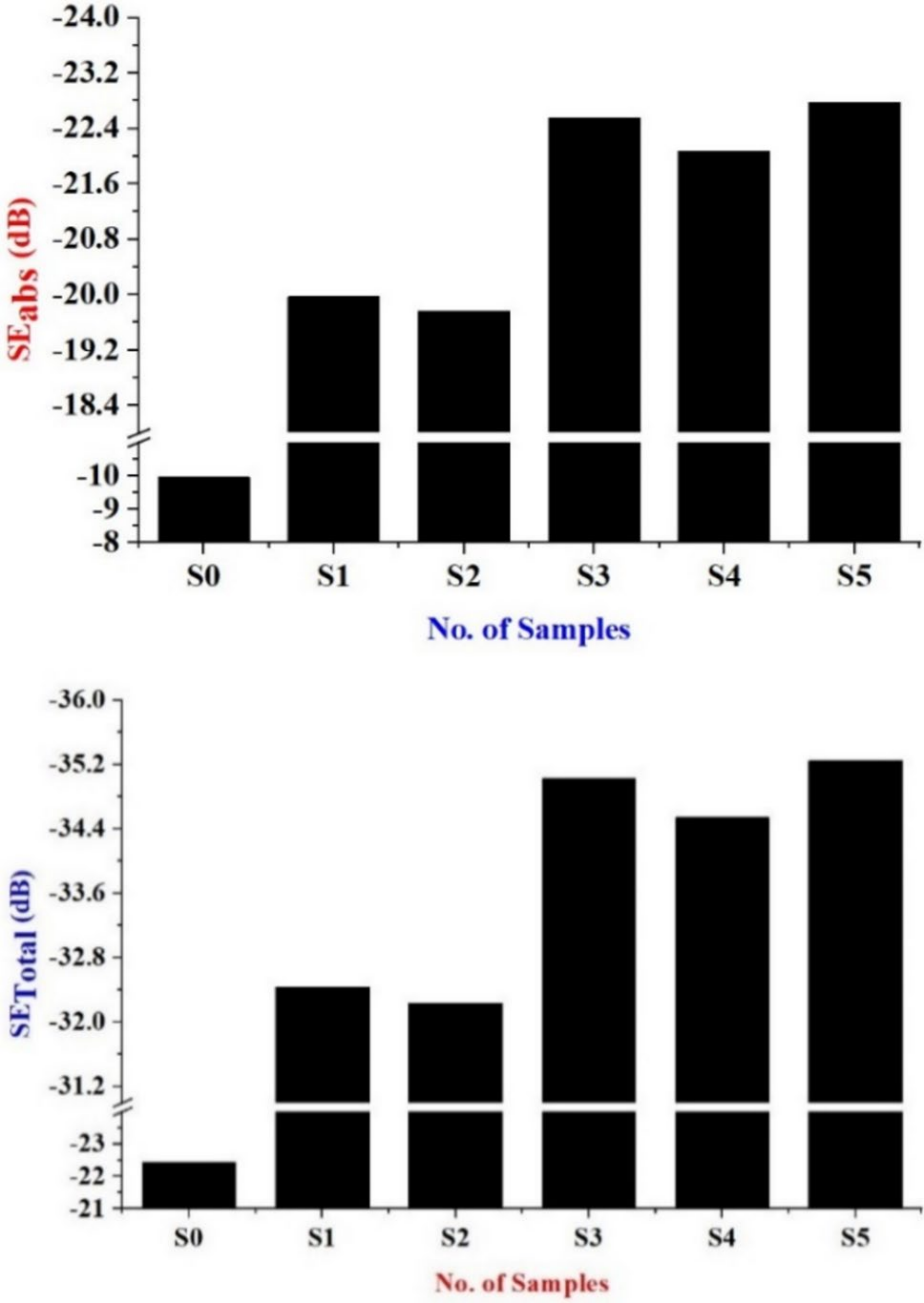
Evaluation of SE_abs_ and SE_Total_ for different bio-epoxy composites.

Table 6 and Fig. 10 show that the shielding effectiveness absorption and total varies with every composition. Still, there is an overall increase in the shielding effectiveness as the amount of filler materials increases. When VMT is added it aids in absorbing and reflecting electromagnetic waves. The structure of VMT with its layered composition can block electromagnetic interference^50^. The total shielding effectiveness of the samples was found using Eqs. (4–9) and the shielding effectiveness of reflection in the free air medium was calculated using Eqs. (4–7) to further confirm that there are no external factors affecting the EMI waves and it is found out to be in the range of −3.775 ~ −12.470 dB^51^. However, when compared to other samples, sample S5 has an excellent EMI shielding effectiveness of −35.2 dB, and compared to the non-reinforced sample, it has an improvement of 57.11%^52^. The negative values of the EMI shielding effectiveness represent the reduction of power when the wave is transmitted through the sample. This value does not come close to replacing existing materials. This is because VMT is a nonconductive material and cannot shield electromagnetic interference as compared to conductive materials. Similar research with composite materials shows the same range of shielding effectiveness^51,53^.

The EMI shielding effectiveness can be calculated by

4 $${\text{Reflection coefficient }}\left( {\text{R}} \right){ = }\left| {{\text{S}}_{{{11}}} } \right|^{{2}}$$

where S_11_ = spectrum parameter in free air medium

5 $${\text{Transmission coefficient }}\left( {\text{T}} \right){ = }\left| {{\text{S}}_{{{21}}} } \right|^{{2}}$$

where S_21_ = spectrum parameter of different WRPU foams

6 $${\text{Absorption coefficient }}\left( {\text{A}} \right){\text{ = 1 - R - T}}$$

7 $$\text{Shielding effectiveness reflection }(\text{SEref}) = -10\times \text{log}(1-R) (dB)$$

8 $$\text{Shielding effectiveness absorption }(\text{SEabs}) =-10\times \text{log}(\frac{T}{1-R}) (dB)$$

9 $${\text{Total shielding effectiveness = SE}}_{{{\text{ref}}}} {\text{ + SE}}_{{{\text{abs}}}} \left( {{\text{dB}}} \right)$$

### Acoustic testing

**Fig. 11:**
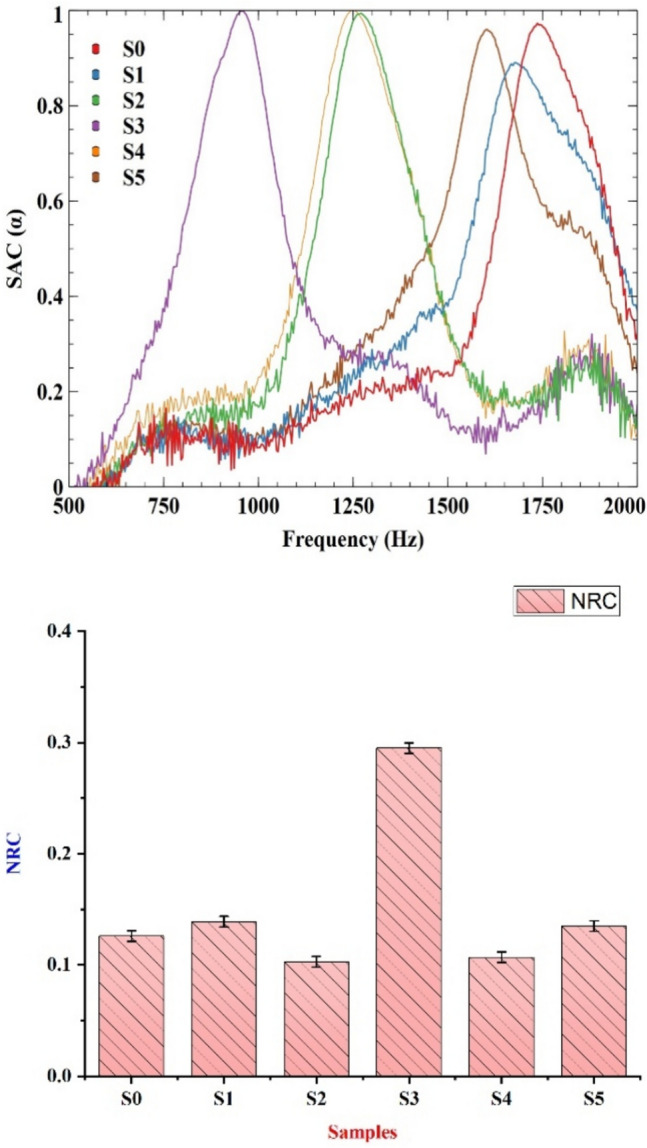
(**a**) Evaluation of SAC values for different frequencies (**b**) Evaluation of NRC for different bio-epoxy composites.

The samples from 0% filler material to 10% filler material are tested for sound absorption properties. From Table 7 and Fig. 11, we can see that the acoustic properties of the samples perform better at a particular frequency compared to other samples. S0 performs better at higher frequencies and other samples perform better at lower frequencies compared to S0 and S3 performs better at the least frequency range of 900–1000 Hz. This variation is due to the viscous losses due to friction between air and particles and friction between particles^54^. The S0 sample performs better at a higher frequency due to its elastic properties that allow the sample’s absorption, whereas the S5 sample performs better at a similar high range due to the friction between the particles tightly packed in the sample. In Fig. 10 the S3 sample performs significantly better at NRC. This is due to the S3 sample performing the best at 1000 Hz frequency with significant differences between other samples thus when calculating for NRC and including all the performances at different frequencies it seems to have higher NRC and thus can be used for general purposes and other samples can be used for specific scenarios where only high or low frequency is involved^42^.

**Table 7:** Evaluation of SAC at the frequency of 250, 500, 1000 and 2000 Hz.

Samples	Frequency (Hz)				
	**250**	**500**	**1000**	**2000**	**NRC**
**S0**	0.05347352741	0.04799482176	0.08299945377	0.3193643174	0.126
**S1**	0.02336518684	0.05733872433	0.1048175601	0.3711126244	0.139
**S2**	0.02053074289	0.06973434191	0.1674546011	0.1555544563	0.103
**S3**	0.08617674015	0.03112557091	0.8896149493	0.1728451148	0.295
**S4**	0.04837766017	0.03958826037	0.2301268946	0.1118836695	0.107
**S5**	0.1254035346	0.06770546762	0.1099757736	0.238251659	0.135

#### Thermal conductivity

**Fig. 12:**
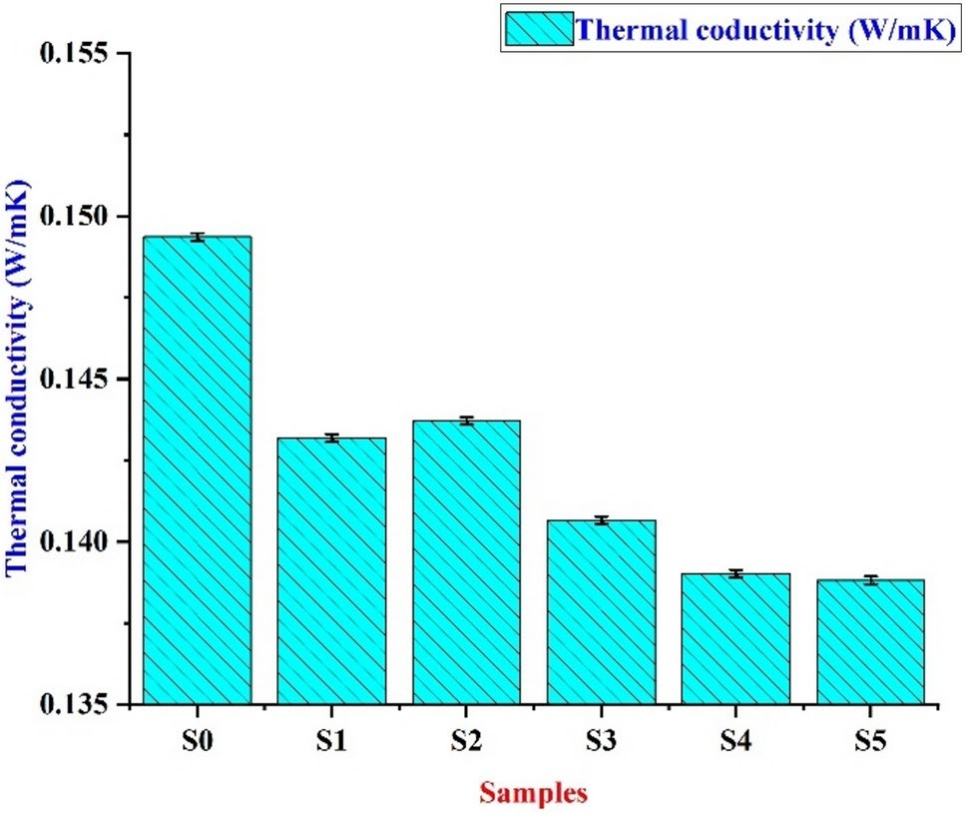
Thermal conductivity for different bio-epoxy composites.

The thermal conductivity test has been tested for all samples. From Table 8 and Fig. 12, we can find that the thermal conductivity sharply decreases when moving from S0 to S1. The reason behind this is the thermal conductivity of Epoxy is high^55^. The sample filled with VMT and WRPU have low thermal conductivity as seen in Table 9. The reason is that both these materials are non-metals. Thus, as the filler amount goes from 2 to 10% the conductivity decreases^56^.

**Table 8:** Thermal conductivity.

**Samples**	**Thermal conductivity** **W/mK**	**Effusivity** **Ws**^**1/2**^**/ Km**^**2**^
S0	0.193673367	305.5417786
S1	0.142936734	304.8619738
S2	0.138816977	301.8542371
S3	0.143190117	305.0417579
S4	0.14294006	304.8643377
S5	0.141266291	303.6618835

**Table 9:** Flammability test.

Sample	T (secs)	L(mm)	Burning rate(mm/min)
S0	57	12.3	12.94736842
S1	76	11	8.684210526
S2	72	9	7.5
S3	69	9	7.826086957
S4	60	11.4	11.4
S5	75	8.5	6.8

#### Flammability test

**Fig. 13:**
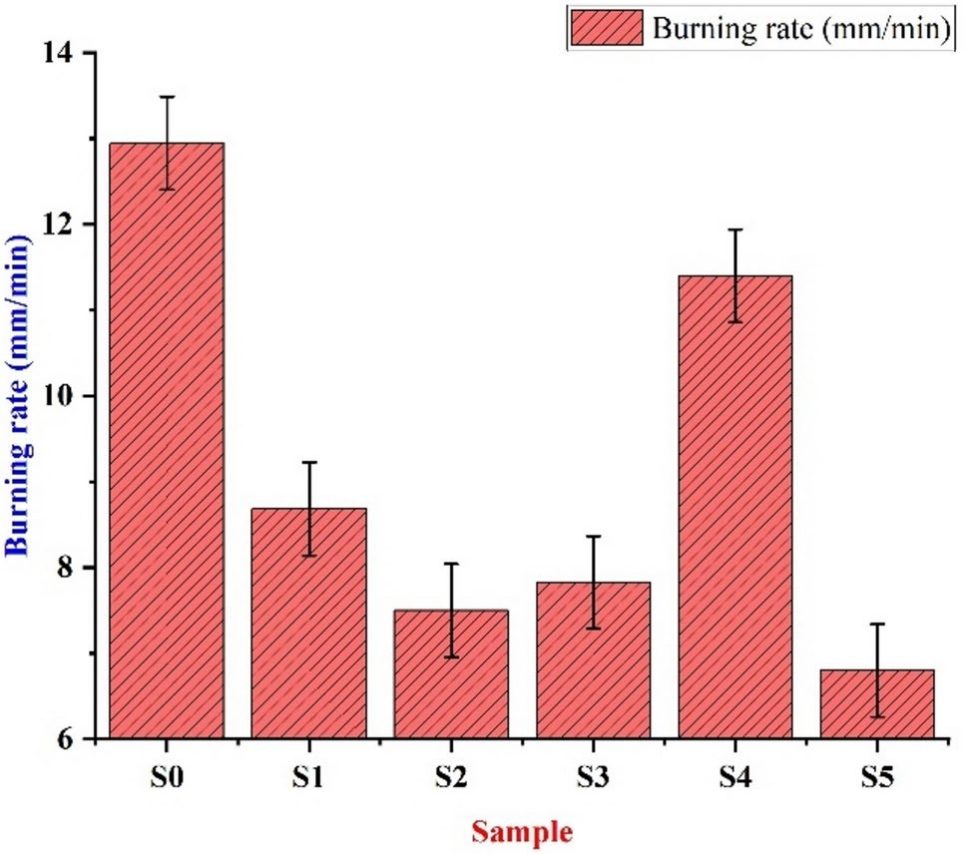
Evaluation of burning rate for different bio-epoxy composites.

The samples are placed in the presto flammability tester vertically under the burner for 10 s and removed from the burner. As the VMT filler is a flame-resistant material, it contributes to reducing the flammability of the composite material. In the beginning, the gradual decrease in the flammability is because the addition of WRPU (5 wt.%) is more than that of VMT (2,4 wt.%). As we know PU burns quickly and forms an expanding layer of carbon. The composite containing 8 wt.% VMT showed better resistance, and it led to the equal dispersibility of VMT and PU components in the epoxy matrix, thereby showing better resistance to flammability. The addition of PU in the epoxy matrix influenced the irregular burning pattern of the samples by reducing the oxygen and the amount of carbon increased in the burning of PU. From Table 9 and Fig. 13, we can see that sample S5 has the lowest burning rate which indicates that the excess amount of VMT prevents homogenization in the matrix, which leads to the formation of an uneven carbon residue layer after combustion, which leads to poor combustion^40^. The comparison of all tests with various materials is shown in Table 10.

**Table 10:** Comparison of all tests with other literature.

Tests	Sample dimensions	Materials	Results	References
**Tensile**	250 × 25x5 mm^3^	Epoxy composite reinforced with Tamarindus	8.13 MPa	^57^
	70 × 15 × 3 mm^3^	50%DPF/epoxy	25.7657 MPa	^58^
	ASTM D638	0.25% GNP for 2 h ultrasonication	50.9 MPa	^59^
	ASTM D2256	Epoxy + 30% jute + 70% glass	329 ± 19.8 MPa	^60^
	ASTM D3039	Epoxy + 5% WRPU + VMT10%	10.4707 N/mm^2^	Our research
**Impact**	ASTM D256	Chitosan (CTS) and date palm (DP) reinforced in epoxy with 40 wt.% DP CTS 20 wt.%	2.70 J/m^2^	^61^
	ASTM D256	50% DPF/epoxy	98.71 J/m^2^	^58^
	ASTM D256	Kevlar 1 layer on top and bottom + 14 layers of E-glass	75 kJ/m^2^	^62^
	ASTM D256	Epoxy + luffa fiber (20 wt.%) + graphene (4 wt.%)	4.352 kJ/m^2^	^63^
	ASTM D5942	Kolon/epoxy/SiC particles	3.21 ± 0.209 J/cm^2^	^64^
	ASTM D256	Epoxy + 5% WRPU + 10% VMT	0.006 kJ/cm^2^	Our research
**Flexural**	-	Sugarcane fiber (SCF) and tamarind seed powder (TSP) reinforced epoxy with 35 wt% SCF/15 wt% TSP	36.543 MPa	^65^
	ASTM D790-02	DOCF15/ 0.2GNP	777 ± 49 MPa	^66^
	ASTM D-790	Epoxy + 30 vol.% glass fiber + 1.5 wt.% clay	232.6 MPa	^67^
	ASTM D7264	25NS-GKFRP (25 wt.% nano-silica in hybrid woven glass/UD kenaf fiber reinforced polymer composites)	126.38 MPa	^68^
	ASTM D790	50%DPF/epoxy	32.64 MPa	^69^
	ASTM D3039	Epoxy + 5wt%WRPU + 10wt%VMT	46.7959 N/mm^2^	Our research
**Dielectric constant**	ASTM D2863-97	fluoro-phosphonium salt(P), melamine(M) reinforced epoxy with 2 wt.% of P and 10 wt.% of M	2.90	^70^
	2 × 2 × 20 mm^3^	hollow glass microspheres(S-HGMs)	1.58	^71^
	-	fluorinated epoxy resin (diglycidol ether of 2,4-bis (1,1,1,3,3,3-hexafluoroisopropyl) fluorobenzene (FB-EP)) with 45.7 wt.% fluorine content was synthesized from fluorobenzene, epichlorohydrin, and hexafluoroacetone	2.23	^72^
	ASTM D150	Epoxy + 5 wt.% WRPU + 10wt.% VMT	1.111	Our research
**EMI Shielding Effectiveness**	-	flame retardant epoxy resins (FREP) reinforced with carbon foam (FREP-CF)	33.5 dB	^73^
	-	2 mm thick epoxy composite reinforced with 5 wt.% SCF along with 1 wt.% MWCNT (CNT-SCF5)	35.4 dB	^74^
	-	addition of vermiculite in 5%, 10% and 15% of total mass of polyurethane material	–20 dB	^75^
	0.7 mm thickness	different weight percentages of magnetite nanoparticles (Fe_3_O_4_) and cost-effective carbon black nanoparticles (CBN)	36.6 dB	^76^
	ASTM D4935	bio-based polyamide 11/poly (lactic acid) composites with various carbon fiber (30% CF)	28 dB	^77^
	ASTM D4935 (50 × 50 × 7 mm3)	Epoxy + 5wt%WRPU + 10wt%VMT	−35.246 dB	Our research
**Acoustic (NRC)**	-	kenaf fiber, carbon black (CB), nickel (Ni), and bamboo charcoal (BC) reinforced epoxy with 14.0 g and 4,6,10 wt.% respectively	0.524	^78^
	-	coal fly ash in nanoparticles varied at 5 – 25 wt. % was employed as reinforcement in the fabrication of epoxy resin composites	0.8072	^79^
	sample thickness of 30 mm	renewable monomer and epoxy as polyol, methylene diphenyl diisocyanate (MDI) as crosslinker	0.4850	^80^
	EN ISO 10,534–2	A binder mixture of an aerial lime type CL-90 S, designated according to the European standard UNE-EN 459–1 with lightweight aggregate vermiculite (V)	0.037 ± 0.002	^81^
	-	pervious concrete includes cement, coarse aggregates with little or no fine aggregate	0.28	^82^
	ASTME 1050–19	Epoxy + 5 wt% WRPU + 10wt% VMT	0.295	Our research
**Thermal conductivity**	-	75 wt% jute 25 wt% snake plant reinforced epoxy composite	0.20 (W/mK)	^83^
	-	APTES((3-aminopropyl) (triethoxysilane)-BNNS(boron nitride nanosheets)/EP 40wt%	5.86 W/mK	^84^
	-	Epoxy + A 6%(alumina) + B 4% BNNs	0.47 W/mK	^85^
	thickness-2.45 mm	Epoxy + jute(plain structure) with 35 + 2% fibre volume fraction	0.25 W/mK	^86^
	ASTM D5470(25 × 25 × 5 mm^3^)	Epoxy + 5wt%WRPU + 10wt%VMT	0.141266291 W/mK	Our research
**Flammability**	ASTM D3801	pure RPU foam, Si-oil, A33, and deionized water were first mixed with polyether polyol	v2 (fail)	^27^
	ASTMD 3801–96	PU + VMT + polyol with isocyanate	5.5 mm/Min v2 (fail)	^40^
	UL 94	carbon foam/epoxy (FREP-CF)	v0 is achieved	^73^
	UL 94	Walnut shell powder reinforced epoxy with pumice powder as filler	v2 (fail)	^87^
	UL 94	Sisal fibre added to epoxy and ammonium phosphate as flame retardant (5wt% to 15 wt%)	v0 is achieved	^88^
	ASTM D3801	Epoxy + 5wt%WRPU + 10wt%VMT	6.8 mm/min V1 (Good)	Our research

## Conclusion

A composite containing VMT and WRPU with bio epoxy as a matrix was fabricated and subjected to investigation on a range of properties. Among the produced specimens the S0 sample performed better at the tensile and flexural properties by 87.07% and 62.18% respectively when compared to sample S5. This is due to its elasticity, which is not feasible to be used for the construction of a component as it can be susceptible to deformation and can pose a threat to the structural integrity of the material. At impact strength, the S5 sample performed 50% better than the S0 sample. The sample S5 performed better at the dielectric constant with a 36.51% improvement from the S0 sample. At electromagnetic interference (EMI) shielding sample S5 performed better than sample S0 by 57.13%. This is due to the absorption properties of VMT. Among the samples tested for thermal conductivity sample S0 had the highest conductive property and S5 had the least conductive property with an improvement of 27.06% when compared to S0. Sample S3 performed better at noise absorption when compared to the S0 sample by 134.13%. The sample S5 has performed the best at dielectric and EMI shielding and can thus be used for battery enclosures and battery casings for electric vehicles. Since the composite also has good acoustic properties, it can be used for engine bay insulation and cabin insulation respectively and since the composite is flame resistant it could aid in the lifespan of these products. The multifunctional capabilities of this material not only improve its performance in these applications but also contribute to the overarching goal of achieving net-zero carbon emissions. Therefore, the developed sustainable material stands out as a highly efficient solution for advancing towards environmentally friendly and energy-efficient technologies.

## Supplementary Information

Supplementary Information 1.

## Data Availability

Data cannot be shared publicly. The data that support the findings of this study are available upon reasonable request from the authors.

## References

[1] Schyns, Z. O. G. & Shaver, M. P. Mechanical recycling of packaging plastics: A review. *Macromol. Rapid Commun.* **42**, 2000415 (2021). 10.1002/marc.202000415 33000883

[2] Rahimi, A. & García, J. M. Chemical recycling of waste plastics for new materials production. *Nat. Rev. Chem.* **1**, 46 (2017).

[3] Thiounn, T. & Smith, R. C. Advances and approaches for chemical recycling of plastic waste. *J. Polym. Sci.* **58**, 1347–1364 (2020).

[4] Shin, S.-R., Kim, H.-N., Liang, J.-Y., Lee, S.-H. & Lee, D.-S. Sustainable rigid polyurethane foams based on recycled polyols from chemical recycling of waste polyurethane foams. *J. Appl. Polym. Sci.* **136**, 47916 (2019).

[5] Deng, Y. et al. Reviewing the thermo-chemical recycling of waste polyurethane foam. *J. Environ. Manage.* **278**, 111527 (2021). 33126201 10.1016/j.jenvman.2020.111527

[6] Gama, N. et al. Recycling of polyurethane scraps via acidolysis. *Chem. Eng. J.* **395**, 125102 (2020).

[7] Cregut, M., Bedas, M., Durand, M.-J. & Thouand, G. New insights into polyurethane biodegradation and realistic prospects for the development of a sustainable waste recycling process. *Biotechnol. Adv.* **31**, 1634–1647 (2013). 23978675 10.1016/j.biotechadv.2013.08.011

[8] Kumar, S. & Krishnan, S. Recycling of carbon fiber with epoxy composites by chemical recycling for future perspective: A review. *Chem. Pap.* **74**, 3785–3807 (2020).

[9] Kemona, A. & Piotrowska, M. Polyurethane recycling and disposal: Methods and prospects. *Polymers*10.3390/polym12081752 (2020). 32764494 10.3390/polym12081752 PMC7464512

[10] Gómez-Rojo, R., Alameda, L., Rodríguez, Á., Calderón, V. & Gutiérrez-González, S. Characterization of polyurethane foam waste for reuse in eco-efficient building materials. *Polymers*10.3390/polym11020359 (2019). 30960343 10.3390/polym11020359 PMC6419407

[11] Capricho, J. C., Fox, B. & Hameed, N. Multifunctionality in Epoxy Resins. *Polym. Rev.* **60**, 1–41 (2020).

[12] Hartwig, A., Arand, M. & Commission, M. A. K. Bisphenol A diglycidyl ether. *The MAK Collection for Occupational Health and Safety* **8**, Doc077 (2023).

[13] Xue, J. et al. A review of properties, production, human exposure, biomonitoring, toxicity, and regulation of bisphenol A diglycidyl ethers and novolac glycidyl ethers. *Environ. Chem. Ecotoxicol.* **4**, 216–230 (2022).

[14] Heinrich, L. A. Future opportunities for bio-based adhesives–advantages beyond renewability. *Green Chem.* **21**, 1866–1888 (2019).

[15] Jing, R. et al. Effect of recycling agents on rheological properties of epoxy bitumen. *Road Mater. Pavement Des.* **23**, 2592–2606 (2022).

[16] Gonçalves, F. A. M. M., Ferreira, P. & Alves, P. Synthesis and characterization of itaconic-based epoxy resin: Chemical and thermal properties of partially biobased epoxy resins. *Polym. (Guildf)* **235**, 124285 (2021).

[17] Gonçalves, F. A. M. M., Santos, M., Cernadas, T., Ferreira, P. & Alves, P. Advances in the development of biobased epoxy resins: Insight into more sustainable materials and future applications. *Int. Mater. Rev.* **67**, 119–149 (2022).

[18] Ma, S. et al. Synthesis and properties of a bio-based epoxy resin with high epoxy value and low viscosity. *ChemSusChem* **7**, 555–562 (2014). 24136894 10.1002/cssc.201300749

[19] Chen, X. et al. Degradable and recyclable bio-based thermoset epoxy resins. *Green Chem.* **22**, 4187–4198 (2020).

[20] Liu, J. et al. Recent development on bio-based thermosetting resins. *J. Polym. Sci.* **59**, 1474–1490 (2021).

[21] Cheng, H.-T., Lee, Y.-S., Liu, H.-C. & Lee, W.-J. The effect of component addition order on the properties of epoxy resin/polyurethane resin interpenetrating polymer network structure. *J. Appl. Polym. Sci.* **138**, 49833 (2021).

[22] Bahramnia, H., Semnani, H. M., Habibolahzadeh, A. & Abdoos, H. Epoxy/polyurethane nanocomposite coatings for anti-erosion/wear applications: A review. *J. Compos. Mater.* **54**, 3189–3203 (2020).

[23] Ma, Z. et al. Effects of Al-based alloy powders on the mechanical behavior, corrosion resistance and infrared emissivity of polyurethane composite coatings. *Colloids Surf A Physicochem. Eng. Asp* **624**, 126782 (2021).

[24] Sleinus, D. et al. Properties of sound absorption composite materials developed using flax fiber, sphagnum moss, vermiculite, and sapropel. *Materials*10.3390/ma16031060 (2023). 36770067 10.3390/ma16031060 PMC9920241

[25] Li, T.-T. et al. Preparation and characteristics of flexible polyurethane foam filled with expanded vermiculite powder and concave-convex structural panel. *J. Mater. Res. Technol.* **12**, 1288–1302 (2021).

[26] Güney, B., Akbulut, F. & Kiliç, H. Investigation of the properties of vermiculite added to the composition of brake pads. *Int. J. Adv. Nat. Sci. Eng. Res.* **7**, 2023 (2023).

[27] Xia, Z. & Wang, Z. Rigid polyurethane/expanded vermiculite/ melamine phenylphosphate composite foams with good flame retardant and mechanical properties. *e-Polymers* **19**, 563–573 (2019).

[28] Kirbaş, İ. Investigation of the internal structure, combustion, and thermal resistance of the rigid polyurethane materials reinforced with vermiculite. *J. Thermoplast. Compos. Mater.* **35**, 1561–1575 (2020).

[29] Barczewski, M. et al. Comprehensive analysis of the influence of expanded vermiculite on the foaming process and selected properties of composite rigid polyurethane foams. *Polymers*10.3390/polym14224967 (2022). 36433094 10.3390/polym14224967 PMC9692458

[30] Madhankumar, S., Yokeshwaran, S., Elavarasan, G., Kannan, M. & Balamurugan, A. Testing of mechanical properties of Aluminum metal matrix composite using vermiculite. *Mater. Today Proc.* **45**, 6974–6978 (2021).

[31] Sevinç, A. H. & Durgun, M. Y. A novel epoxy-based composite with eggshell, PVC sawdust, wood sawdust and vermiculite: An investigation on radiation absorption and various engineering properties. *Constr. Build Mater.* **300**, 123985 (2021).

[32] Koksal, F., Sahin, Y. & Gencel, O. Influence of expanded vermiculite powder and silica fume on properties of foam concretes. *Constr. Build Mater.* **257**, 119547 (2020).

[33] Bhingare, N. H., Prakash, S. & Jatti, V. S. A review on natural and waste material composite as acoustic material. *Polym Test* **80**, 106142 (2019).

[34] Führ, G., Masuero, A. B., Pagnussat, D. T. & Menna Barreto, M. F. F. Impact sound attenuation of subfloor mortars made with exfoliated vermiculite and chrome sawdust. *Appl. Acoust.* **174**, 107725 (2021).

[35] Hanken, R. B. L. et al. Effect of natural and expanded vermiculite clays on the properties of eco-friendly biopolyethylene-vermiculite clay biocomposites. *Compos. B Eng.* **175**, 107184 (2019).

[36] Aniśko, J. et al. The relationship between a rotational molding processing procedure and the structure and properties of biobased polyethylene composites filled with expanded vermiculite. *Materials*10.3390/ma15175903 (2022). 36079285 10.3390/ma15175903 PMC9457396

[37] Kim, S. et al. Amplifying the dielectric constant of shellac by incorporating natural clays for organic field effect transistors (OFETs). *Turk J. Chem.* **47**, 1169–1182 (2023). 38173751 10.55730/1300-0527.3603 PMC10762868

[38] Tserpes, K., Tzatzadakis, V. & Bachmann, J. Electrical conductivity and electromagnetic shielding effectiveness of bio-composites. *J. Compos. Sci.*10.3390/jcs4010028 (2020).

[39] Nabipour, H., Wang, X., Song, L. & Hu, Y. A fully bio-based coating made from alginate, chitosan and hydroxyapatite for protecting flexible polyurethane foam from fire. *Carbohydr. Polym.* **246**, 116641 (2020). 32747276 10.1016/j.carbpol.2020.116641

[40] Alves, L. R. P. S. T. et al. Polyurethane/vermiculite foam composite as sustainable material for vertical flame retardant. *Polym. (Basel)* **14**(8), 377 10.3390/polym14183777 (2022). 10.3390/polym14183777 PMC9504044 36145923

[41] Sabu, M., Bementa, E., Ruban, J. V. & Mon, Y. S. G. A novel analysis of the dielectric properties of hybrid epoxy composites. *Adv. Compos. Hybrid Mater.* **3**, 325–335 (2020).

[42] Chen, L. et al. Preparation and characterization of the eco-friendly chitosan/vermiculite biocomposite with excellent removal capacity for cadmium and lead. *Appl. Clay Sci.* **159**, 74–82 (2018).

[43] Zhou, X. et al. Preparation and characterization of waterborne polyurethane/cellulose nanocrystal composite membrane from recycling waste paper. *J. Renew. Mater.* **8**(6), 631 10.32604/jrm.2020.010176 (2020).

[44] Marcos, C. & Rodríguez, I. Expansibility of vermiculites irradiated with microwaves. *Appl. Clay Sci.* **51**, 33–37 (2011).

[45] Feng, J., Liu, M., Mo, W. & Su, X. Heating temperature effect on the hygroscopicity of expanded vermiculite. *Ceram. Int.* **47**, 25373–25380 (2021).

[46] He, P. et al. Mechanochemistry: An efficient way to recycle thermoset polyurethanes. *Polym. (Basel)*10.3390/polym14163277 (2022). 10.3390/polym14163277 PMC9412547 36015532

[47] Gupta, U. S., Dhamarikar, M., Dharkar, A., Tiwari, S. & Namdeo, R. Study on the effects of fibre volume percentage on banana-reinforced epoxy composite by finite element method. *Adv. Compos. Hybrid Mater.* **3**, 530–540 (2020).

[48] Rudresh, B. M., Kumar, B. N. R. & Madhu, D. Combined effect of micro- and nano-fillers on mechanical, thermal, and morphological behavior of glass–carbon PA66/PTFE hybrid nano-composites. *Adv. Compos. Hybrid Mater.* **2**, 176–188 (2019).

[49] Nagaraja, S., Anand, P. B., Mahadeva Naik, R. N. & Gunashekaran, S. Effect of aging on the biopolymer composites: Mechanisms, modes and characterization. *Polym. Compos.* **43**, 4115–4125 (2022).

[50] Katheria, A. et al. Highly flexible EMA/Fe3O4@g-C3N4 composite for thermal control and EMI shielding application. *Colloids Surf. A Physicochem. Eng. Asp.* **700**, 134756 (2024).

[51] Nivedhitha, D. M. et al. Biopolymer-coated composites for enhanced dielectric and electromagnetic interference shielding applications - a green initiative. *Mater. Res. Express* **10**, 105013 (2023).

[52] Hong, J. et al. Regulated orientation and exfoliation of flaky fillers by close packing structures in polymer composites for excellent thermal conduction and EMI shielding. *Compos. B Eng.* **275**, 111357 (2024).

[53] Yang, W. et al. Robust and mechanically and electrically self-healing hydrogel for efficient electromagnetic interference shielding. *ACS Appl. Mater. Interfaces* **10**, 8245–8257 (2018). 29381055 10.1021/acsami.7b18700

[54] Ghofrani, M., Ashori, A. & Mehrabi, R. Mechanical and acoustical properties of particleboards made with date palm branches and vermiculite. *Polym. Test* **60**, 153–159 (2017).

[55] Struzziero, G., Remy, B. & Skordos, A. A. Measurement of thermal conductivity of epoxy resins during cure. *J. Appl. Polym. Sci.* **136**, 47015 (2019).

[56] Dev, B. et al. Recent progress in thermal and acoustic properties of natural fiber reinforced polymer composites: Preparation, characterization, and data analysis. *Polym. Compos.* **44**, 7235–7297 (2023).

[57] Subithalini, K., Pugazhenthi, R., Sridhar, R. & Gnanavel, C. Comparison of mechanical stability and morphology study between tree fibres and shrub fibres reinforced epoxy bio-composites. *Mater. Today Proc.*10.1016/j.matpr.2024.06.010 (2024).

[58] Saba, N., Alothman, O. Y., Almutairi, Z., Jawaid, M. & Ghori, W. Date palm reinforced epoxy composites: Tensile, impact and morphological properties. *J. Mater. Res. Technol.* **8**, 3959–3969 (2019).

[59] Kilic, U., Sherif, M. M. & Ozbulut, O. E. Tensile properties of graphene nanoplatelets/epoxy composites fabricated by various dispersion techniques. *Polym. Test.* **76**, 181–191 (2019).

[60] Mostafa, N. H. Tensile and fatigue properties of Jute-Glass hybrid fibre reinforced epoxy composites. *Mater. Res. Express* **6**, 85102 (2019).

[61] Sarmin, S. N. et al. Enhancing the properties of date palm fibre reinforced bio-epoxy composites with chitosan – Synthesis, mechanical properties, and dimensional stability. *J. King Saud Univ. Sci.* **35**, 102833 (2023).

[62] Alagumalai, V. et al. Impact response and damage tolerance of hybrid glass/kevlar-fibre epoxy structural composites. *Polymers*10.3390/polym13162591 (2021). 34451131 10.3390/polym13162591 PMC8400536

[63] Ashok, K. G. & Kalaichelvan, K. Mechanical, ballistic impact, and water absorption behavior of luffa/graphene reinforced epoxy composites. *Polym. Compos.* **41**, 4716–4726 (2020).

[64] Obradović, V., Simić, D., Sejkot, P., Machalická, K. V. & Vokáč, M. Moisture absorption characteristics and effects on mechanical properties of Kolon/epoxy composites. *Curr. Appl. Phys.* **26**, 16–23 (2021).

[65] Girimurugan, R., Shilaja, C., Mayakannan, S., Rajesh, S. & Aravinth, B. Experimental investigations on flexural and compressive properties of epoxy resin matrix sugarcane fiber and tamarind seed powder reinforced bio-composites. *Mater. Today Proc.* **66**, 822–828 (2022).

[66] Srivastava, A. K., Gupta, V., Yerramalli, C. S. & Singh, A. Flexural strength enhancement in carbon-fiber epoxy composites through graphene nano-platelets coating on fibers. *Compos. B Eng.* **179**, 107539 (2019).

[67] Rafiq, A. & Merah, N. Nanoclay enhancement of flexural properties and water uptake resistance of glass fiber-reinforced epoxy composites at different temperatures. *J. Compos. Mater.* **53**, 143–154 (2018).

[68] Sapiai, N., Jumahat, A., Jawaid, M., Midani, M. & Khan, A. Tensile and flexural properties of silica nanoparticles modified unidirectional kenaf and hybrid glass/kenaf epoxy composites. *Polymers*10.3390/polym12112733 (2020). 33217951 10.3390/polym12112733 PMC7698630

[69] Gheith, M. H. et al. Flexural, thermal and dynamic mechanical properties of date palm fibres reinforced epoxy composites. *J. Mater. Res. Technol.* **8**, 853–860 (2019).

[70] Huang, Z. et al. Fire safe epoxy composite with low dielectric properties from a combination of fluoro-phosphonium salt, melamine and copper hydroxystannate. *Polym. Degrad. Stab.* **202**, 110033 (2022).

[71] Wu, B. et al. Epoxy-matrix composite with low dielectric constant and high thermal conductivity fabricated by HGMs/Al2O3 co-continuous skeleton. *J. Alloys Compd.* **869**, 159332 (2021).

[72] Li, J., Chen, M. & Wang, Y. Preparation and properties of a fluorinated epoxy resin with low dielectric constant. *J. Appl. Polym. Sci.* **139**, 52132 (2022).

[73] Guo, W. et al. Multifunctional epoxy composites with highly flame retardant and effective electromagnetic interference shielding performances. *Compos. B Eng.* **192**, 107990 (2020).

[74] Parameswarreddy, G., Yadam, Y. R., Arunachalam, K., Sarathi, R. & Suematsu, H. Investigation on the enhancement of electromagnetic shielding with efficient use of short carbon fiber in MWCNT-epoxy nanocomposites. *Polym. Compos.* **44**, 1522–1533 (2023).

[75] Wang, J. et al. Lightweight and flexible polysulfonamide/polyurethane-CNTs@Ag fiber paper for electromagnetic interference shielding. *Sustain. Mater. Technol.* **41**, e01088 (2024).

[76] Fallah, R., Hosseinabadi, S. & Pourtaghi, G. Influence of Fe3O4 and carbon black on the enhanced electromagnetic interference (EMI) shielding effectiveness in the epoxy resin matrix. *J. Environ. Health Sci. Eng.* **20**, 113–122 (2022). 35669823 10.1007/s40201-021-00759-x PMC9163220

[77] Durmaz, B. U., Salman, A. O. & Aytac, A. Electromagnetic interference shielding performances of carbon-fiber-reinforced PA11/PLA composites in the X-Band frequency range. *ACS Omega* **8**, 22762–22773 (2023). 37396289 10.1021/acsomega.3c01656 PMC10308563

[78] Selvaraj, V. K. et al. Influence of bio-based kenaf polymer composites on mechanical and acoustic properties for futuristic applications: An initiative towards net-zero carbon emissions. *Polym. Test.* **134**, 108409 (2024).

[79] Durowaye, S., Sekunowo, O., Tiamiyu, J. & Popoola, S. Assessing sound-muffling characteristics of fly-ash nano-particle reinforced epoxy resin composites. *Eskişehir Tech. Univ. J. Sci. Technol. A – Appl. Sci. Eng.* **21**, 514–524 (2020).

[80] Wahab, H. A., Rus, A. Z. M., Moen, A. T., Ngadimon, K. N. & Noor, F. M. Optimization of acoustical properties polyurethane (PU) wood fibres foam composites. *IOP Conf. Ser. Mater. Sci. Eng.* **824**, 12016 (2020).

[81] Palomar, I. & Barluenga, G. Acoustic assessment of multiscale porous lime-cement mortars. *Materials*10.3390/ma16010322 (2023). 10.3390/ma16010322 PMC9822032 36614661

[82] Tie, T. S. et al. Sound absorption performance of modified concrete: A review. *J. Build. Eng.* **30**, 101219 (2020).

[83] Dev, B. et al. Mechanical and thermal properties of unidirectional jute/snake plant fiber-reinforced epoxy hybrid composites. *Ind. Crops. Prod.* **218**, 118903 (2024).

[84] Liu, Z., Li, J. & Liu, X. Novel functionalized BN Nanosheets/Epoxy composites with advanced thermal conductivity and mechanical properties. *ACS Appl. Mater. Interfaces* **12**, 6503–6515 (2020). 31933354 10.1021/acsami.9b21467

[85] Wang, Z. et al. Simultaneously enhanced dielectric properties and through-plane thermal conductivity of epoxy composites with alumina and boron nitride nanosheets. *Sci. Rep.* **11**, 2495 (2021). 33510309 10.1038/s41598-021-81925-x PMC7844292

[86] Mishra, R. et al. Bio-composites reinforced with natural fibers: comparative analysis of thermal, static and dynamic-mechanical properties. *Fibers Polym.* **21**, 619–627 (2020).

[87] Koyunucu, M. & Ulay, G. Thermal and flammability behavior of walnut shell reinforced epoxy composites. *Polímeros* **33**(2), 1–6 10.1590/0104-1428.20230018 (2023).

[88] Pal, D. B., Sinha, S. & Prasad, N. Effect of ammonium phosphate on the thermal and flammability behaviour of sisal/epoxy composite. *Int. J. Adv. Eng. Sci. Technol. Res* **6**, 21–23 (2020).

