# Supplementary figures and images for: Sustainable development of bioepoxy composites reinforced with recycled rigid polyurethane foam for mechanical, thermal, acoustic, and electromagnetic applications in a circular economy approach
Source: Sci Rep. 2025 Mar 10;15:8295. doi: 10.1038/s41598-025-91273-9 (PMC11894226; doi:10.1038/s41598-025-91273-9)

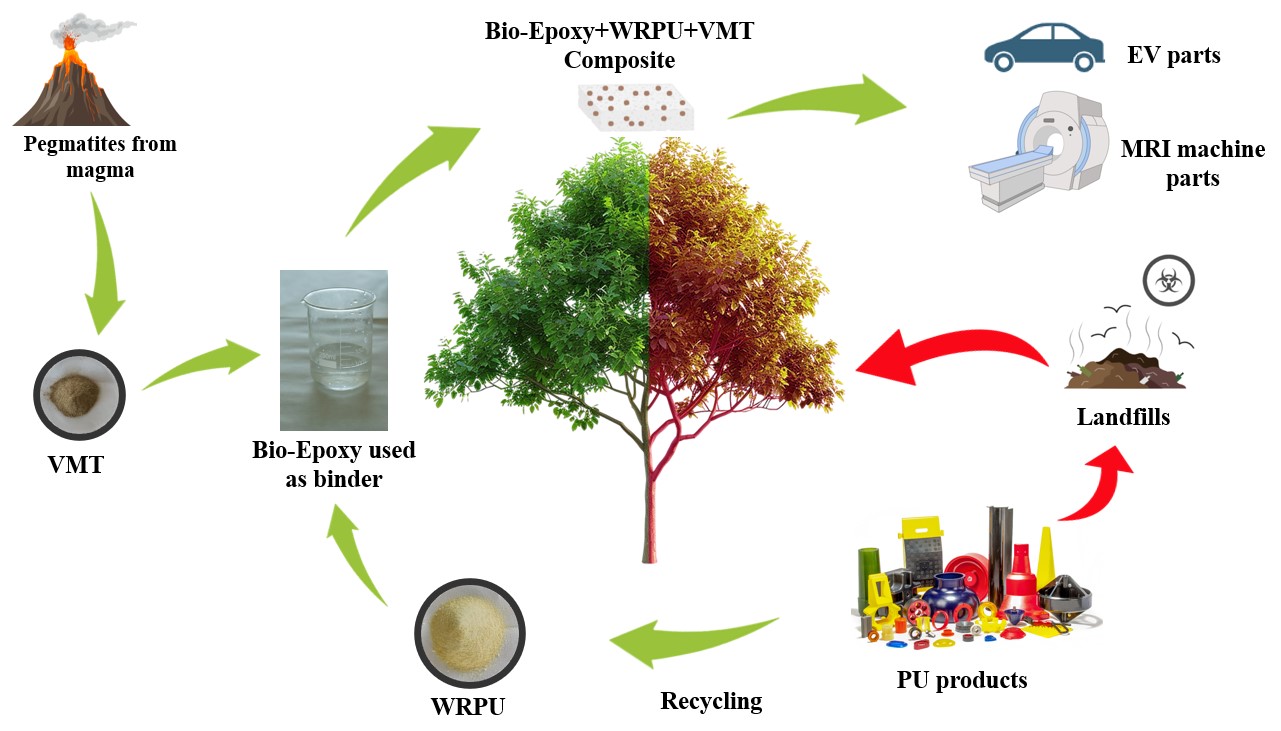

Supplement: Supplementary file 1 — Supplementary Information 1. [file 41598_2025_91273_MOESM1_ESM.jpg]
